# Sugar conundrum in plant–pathogen interactions: roles of invertase and sugar transporters depend on pathosystems

**DOI:** 10.1093/jxb/erab562

**Published:** 2022-02-01

**Authors:** Yong-Hua Liu, You-Hong Song, Yong-Ling Ruan

**Affiliations:** 1 School of Horticulture, Hainan University, Haikou, China; 2 Key Laboratory for Quality Regulation of Tropical Horticultural Crops of Hainan Province, Haikou, China; 3 Innovation Cluster of Crop Molecular Biology and Breeding, Anhui Agricultural University, Hefei, China; 4 School of Agronomy, Anhui Agricultural University, Hefei, China; 5 School of Environmental and Life Sciences, The University of Newcastle, Callaghan, NSW, Australia; 6 Northwest Agriculture and Forestry University, China

**Keywords:** Bacteria, fungi, invertase, pathogen, STP, sugar metabolism, sugar transport, sugar signaling, SWEET, virus

## Abstract

It has been increasingly recognized that CWIN (cell wall invertase) and sugar transporters including STP (sugar transport protein) and SWEET (sugar will eventually be exported transporters) play important roles in plant–pathogen interactions. However, the information available in the literature comes from diverse systems and often yields contradictory findings and conclusions. To solve this puzzle, we provide here a comprehensive assessment of the topic. Our analyses revealed that the regulation of plant–microbe interactions by CWIN, SWEET, and STP is conditioned by the specific pathosystems involved. The roles of CWINs in plant resistance are largely determined by the lifestyle of pathogens (biotrophs versus necrotrophs or hemibiotrophs), possibly through CWIN-mediated salicylic acid or jasmonic acid signaling and programmed cell death pathways. The up-regulation of SWEETs and STPs may enhance or reduce plant resistance, depending on the cellular sites from which pathogens acquire sugars from the host cells. Finally, plants employ unique mechanisms to defend against viral infection, in part through a sugar-based regulation of plasmodesmatal development or aperture. Our appraisal further calls for attention to be paid to the involvement of microbial sugar metabolism and transport in plant–pathogen interactions, which is an integrated but overlooked component of such interactions.

## Teaser

Analyses of diverse plant species and pathogens indicate that the specific roles of invertases and sugar transporters in plant–pathogen interactions depend on the lifestyle and infection strategies of pathogens.

## Introduction

Significant progress has been made in understanding plant–pathogen interactions, and this understanding is essential for achieving sustainable crop production. Upon infection by pathogens including bacteria, fungi and viruses, plants activate various defense systems to prevent or slow down pathogen proliferation (Berger *et al.*, 2007). These defense responses may take place at multiple levels, ranging from cell wall thickening and callose deposition to the generation of reactive oxygen species and immunity-related phytohormones, namely salicylic acid (SA), jasmonic acid (JA), and ethylene (ET), as well as *de novo* biosynthesis of defense-related proteins and secondary metabolites such as phytoalexins and phenolics (Proels and Hückelhoven, 2014; Rojas *et al.*, 2014; Tauzin and Giardina, 2014). To meet the intensive demand for energy, carbon (C) nutrients, and reducing agents that are required for defense responses, the plant primary metabolism is reprogrammed during infection. This reprogramming may include, for example, reduced photosynthesis, increased respiration, and altered nitrogen (N), lipid, and carbohydrate metabolism (reviewed by Bolton, 2009; Rojas *et al.*, 2014). To this end, sugars are the main source of energy and C skeletons for plant defense responses (Morkunas and Ratajczak, 2014). Pathogen-induced shutdown of leaf photosynthesis often leads to source-to-sink transition of the infected tissues (Bolton, 2009). The metabolic shift from source to sink status mandates sugar import into the infected tissue from adjacent or distal healthy source leaves, via long-distance translocation, in the form of sucrose (Suc).

Unsurprisingly, accumulating evidence is revealing vital roles of Suc metabolism and transport in plant resistance or adaptation to biotic stresses (Koch, 2004; Ruan, 2014; Wang and Ruan, 2016). It has long been noted that plant–pathogen interactions are significantly influenced by the amounts of soluble sugar in the host plants. For example, an increase in the sugar content in tomato leaves enhanced resistance to foliar disease target spot caused by *Alternaria solani* (Horsfall and Dimond, 1957). Similarly, pre-treatment of rice and Arabidopsis plants with exogenous Suc, glucose (Glc), or fructose (Fru) increased resistance to the fungus *Magnaporthe oryzae* and the bacterial pathogen *Pseudomonas syringae* pv. *tomato* DC3000 (*Pst* DC3000), respectively (Gómez-Ariza *et al.*, 2007; Qian *et al.*, 2015), indicating an intimate involvement of sugars in the plant–pathogen interaction. 

Theoretically, sugar metabolism and transport may modulate plant–pathogen interactions in several ways. First, sugars provide C nutrients and energy to fuel defense responses. There is a dramatic increase in sugar demand for defense responses including cell wall strengthening, biosynthesis of phytoalexins, and induction of defense-related proteins, for example, pathogenesis-related (PR) proteins (Berger *et al.*, 2007). Secondly, accumulated soluble sugars (especially hexose) themselves may activate the expression of defense-related genes, such as various PR genes, through sugar signaling (Herbers *et al.*, 2000; Sonnewald *et al.*, 2012; Gebauer *et al.*, 2017; Ru *et al.*, 2017). Lastly, soluble sugars, especially those in the extracellular matrix, often serve as a source of C for the growth of pathogens, thereby increasing their virulence (Pommerrenig *et al.*, 2020). Thus, sugars could exert positive or negative roles in plant defense against pathogen infection, depending on the outcome of the ‘tug of war’ between pathogens and plant cells competing for sugar resources.

The apoplasm of plant cells forms the frontier for fighting pathogens upon infection. This cell wall matrix is often enriched in nutrients including sugars, and thus is the main battlefield for pathogens to compete with the host cells for the resources required for colonization (Naseem *et al.*, 2017). The degradation of Suc in the apoplasm and subsequent transport of sugars across plasma membranes (PMs) determine not only the concentrations and composition of sugars in the apoplasm (Bezrutczyk *et al.*, 2018), but also the partitioning of organic C between the host and the pathogen, and hence the outcome of the plant–pathogen interaction (Lemonnier *et al.*, 2014). Some aspects of the involvement of sugar metabolism, transport, and signaling in plant–pathogen interactions have been recently reviewed (Naseem *et al.*, 2017; El Kasmi *et al.*, 2018; Pommerrenig *et al.*, 2020). Those analyses highlighted that, to colonize plants successfully, bacterial and fungal pathogens typically hijack plant sugar transport systems to export sugars into the apoplasmic space to fuel their growth, mainly by inducing the expression of host SWEETs (Sugars Will Eventually be Exported Transporters), a class of energy-independent uniporters for moving sugars across membranes (Chen *et al.*, 2010). As a countermeasure, host cells up-regulate the expression or activity of energy-dependent STPs (Sugar Transport Proteins) and SUTs (SUgar Transporters) to retrieve the apoplasmic sugars back into the cytosol of the plant cells, thereby starving the pathogens in many circumstances (Naseem *et al.*, 2017; El Kasmi *et al.*, 2018, and references therein). Pommerrenig *et al* (2020) pointed out that STP, but not SUT, is likely the major sugar transporter responsible for the reuptake of apoplasmic sugar to minimize bacterial infection. Another countermeasure is the up-regulation of host cell wall invertase (CWIN) upon pathogen infection. CWIN-derived hexoses could act as signaling molecules to activate plant defense responses such as oxidative burst, hypersensitive response, cell wall biosynthesis, the production of secondary metabolites, and alteration of the circadian clock and stomatal aperture (Proels and Hückelhoven, 2014; Tauzin and Giardina, 2014; and references therein). 

Despite the progress outlined above, many questions remain. First, current available studies show that CWINs, SWEETs, and STPs appear to play contradictory or even contrasting roles in plant–pathogen interactions in different systems (as discussed in the following sections). The underlying basis and implications remain elusive. Second, while much attention has been paid to the roles of CWIN and sugar transporters in the host response to bacterial and fungal attack (e.g. Pommerrenig *et al.*, 2020), the potential roles of these proteins in the response to viral infection seem to have been overlooked. Third, little is known about the roles of pathogen-originating CWINs and sugar transporters in plant–pathogen interactions. Here, we address these issues by assessing the relevant information available and providing likely scenarios and insights into this sugar conundrum in plant–pathogen interactions. We then propose several perspectives for future studies to improve our understanding of sugar-mediated plant defense.

## Activation and fueling of plant defenses by CWIN

In higher plants, sucrose synthase (Sus) and invertase (INV) are the two classes of enzymes that degrade Suc into hexose. Sus is a glycosyl transferase that reversibly converts Suc in the presence of UDP into UDP-Glc and Fru, whereas INV irreversibly hydrolyzes Suc into Glc and Fru. Based on their subcellular locations, INVs are further classified into cell wall invertase (CWIN), vacuolar invertase (VIN), and cytoplasmic invertase (CIN) (Sturm, 1999; Wan *et al.*, 2018). To date, studies on the INV-mediated regulation of plant–pathogen interactions have been predominantly conducted on CWINs, as detailed below. This is not surprising, since the apoplasm is the main site of the battle between plant cells and pathogens competing for sugar resources, and also a major cellular site eliciting sugar signaling for defense and development (Naseem *et al.*, 2017; Liao *et al.*, 2020).

In general, CWIN activity is induced or enhanced during pathogen infection (Joosten *et al.*, 1990; Essmann *et al.*, 2008; Bonfig *et al.*, 2010), indicating its positive role in defense. Under pathogen attack, plants experience an increased demand for sugar to trigger and sustain defense responses, which are energy costly (Engelsdorf *et al.*, 2013 and references therein). The increased sugar level found in infected tissues is largely attributed to the induction of CWIN activity, which enhances the sink strength of infected tissues (Proels and Hückelhoven, 2014; Tauzin and Giardina, 2014). The build-up of soluble sugar may potentiate the local defense at the infection site. In addition, CWIN-derived hexose may activate defense responses via signaling. It has been suggested that some unknown sugar receptors localized on the PM likely sense apoplasmic hexose to prime plant defense responses (Bezrutczyk *et al.*, 2018), although such a receptor is yet to be identified.

## The dual role of CWIN in plant–pathogen interactions and its underlying basis

A number of studies have shown positive roles of CWINs in defense against pathogens. The expression of yeast INV in leaf apoplasm increased the resistance of tobacco to potato virus Y, probably owing to the elevated hexose concentration in the leaves activating systemic acquired resistance, including the up-regulation of defense-related genes and peroxidase activities as well as enhanced synthesis of callose and SA (Herbers *et al.*, 1996). More recently, CWIN-overexpressing rice lines had increased concentrations of Suc, Glc, and Fru in leaves and displayed enhanced resistance to the bacterial pathogen *Xanthomonas oryzae* pv. *oryzae* (Xoo) and the fungal pathogen *M. oryzae* through increasing cell wall thickness and activating the expression of PR genes (Sun *et al.*, 2014). Consistently, down-regulation of CWIN reduced the resistance of tobacco to the oomycete *Phytophthora nicotianae* due to decreases in callose deposition, H_2_O_2_ accumulation, and hypersensitive cell death (Essmann *et al.*, 2008).

However, emerging evidence also shows that CWIN could play negative roles in several pathosystems. For instance, silencing of the CWIN gene *LIN8* in tomato delayed the development of disease symptoms in leaves infected by the bacterial pathogen *Xanthomonas campestris* pv. *vesicatoria* (Xcv), although bacterial growth *in planta* remained unchanged in the transgenic tomato plants (Kocal *et al.*, 2008). Here, assessment of plant tolerance to pathogens is generally based on the development of symptoms, rather than the growth or number of pathogenic microbes *in planta* (Kranz, 1988). Thus, it can be concluded that the silencing of *LIN8* expression enhanced tomato tolerance to Xcv. Likewise, reduced CWIN activity achieved through overexpression of a CWIN inhibitor gene increased the tolerance of Arabidopsis to the fungal pathogen *Plasmodiophora brassicae,* which causes clubroot symptoms (Siemens *et al.*, 2011). The observations discussed above indicate the contrasting roles of CWINs in plant defense.

Plant pathogens can be classified according to their lifestyles into two groups, biotrophs and necrotrophs/hemibiotrophs, which feed on living and dead plant tissues, respectively (Glazebrook, 2005, Spanu, 2012). Alterations in metabolism or signaling in the host appear to have opposite effects on resistance to these two groups of pathogens. For example, the barley mutant *albostrians*, which has pale leaves due to blocked chloroplast development, showed decreased resistance to the hemibiotrophic fungus *Bipolaris sorokiniana* (Schäfer *et al.*, 2004) but increased resistance to the biotrophic powdery mildew fungus *Blumeria graminis* (Jain *et al.*, 2004). Similarly, the functional loss of MLO, a PM-localized protein that interacts with cytoplasmic calmodulin, led to broad-spectrum resistance in barley to all known isolates of biotrophic powdery mildew fungi, but increased susceptibility to hemibiotrophic *Magnaporthe grisea* and necrotrophic *B. sorokiniana* (Jarosch *et al.*, 1999; Kumar *et al.*, 2001; Panstruga, 2005).

On the basis of evaluation of a large number of cases of plant responses to different pathosystems, it appears clear that that CWINs could exert different roles in plant defense depending on the lifestyle of the pathogen involved. As summarized in Table 1, CWIN enhances plant resistance to hemibiotrophic pathogens such as the oomycete *P. nicotianae* (Essmann *et al.*, 2008), the bacterial pathogen Xoo, and the fungal pathogen *M. oryzae* (Sharma *et al.*, 2013; Sun *et al.*, 2014), but reduces host resistance to biotrophic pathogens including the fungal pathogen *P. brassicae* (Siemens *et al.*, 2011) and the bacterial pathogen Xcv (Tamir-Ariel *et al.*, 2007; Kocal *et al.*, 2008). The model could be validated through further studies, by, for example, testing the roles of CWIN in response to biotrophic and necrotrophic/hemibiotrophic pathogens simultaneously using genome editing to knock out the CWIN gene. It should be pointed out that the model does not appear to be applicable to viruses, which exert their virulence in a different way from pathogenic bacteria and fungi and thus will be discussed separately in this review. Similar to CWIN-mediated C metabolism, N metabolism also shows contradictory roles in plant defense, depending on the lifestyle of the pathogen (as reviewed by Seifi *et al.*, 2013). For instance, a supply of high N to hydroponically cultivated tomato reduced susceptibility to the necrotrophic fungus *Botrytis cinerea* but increased susceptibility to the biotrophic powdery mildew fungus *Oidium lycopersicum* (Hoffland *et al.*, 2000). It has been hypothesized that an earlier and more dramatic induction of CWIN increases plant resistance via hexose signaling that triggers defense responses, whereas a late and moderate induction of CWIN benefits pathogen development through increasing the supply of sugar to the microbes (Scharte *et al.*, 2005; El Kasmi *et al.*, 2018). However, this cannot explain why the roles of CWIN in defense vary with the lifestyle of the pathogen. Below, we propose two possible scenarios underlying the contrasting roles of CWIN in plant–pathogen interactions.

**Table 1.** Contrasting roles of cell wall invertase (CWIN) in plant resistance to pathogens with different lifestyles

Pathogen	Lifestyle	Role in plant defense	Host	Reference
*Phytophthora nicotianae* (oomycete)	Hemibiotrophic	Positive	Tobacco source leaf	Essmann *et al.* (2008)
*Xanthomonas oryzae* pv. *oryzae* (Xoo; bacterium)	Hemibiotrophic	Positive	Rice source leaf	Sun *et al.* (2014)
*Magnaporthe oryzae* (fungus)	Hemibiotrophic	Positive	Rice source leaf	Sun *et al.* (2014)
*Plasmodiophora brassicae* (fungus)	Biotrophic	Negative	Arabidopsis root	Siemens *et al.* (2011)
*Xanthomonas campestris* pv. *vesicatoria* (Xcv; bacterium)	Biotrophic	Negative	Tomato source leaf	Kocal *et al.* (2008)
Potato virus Y (virus) ^a^	Biotrophic	Positive	Tobacco source leaf	Herbers *et al.* (1996)

CWINs may employ unique plasmodesmata-related mechanisms to regulate plant resistance to virus.

First, phytohormones, especially SA and JA, may be involved in CWIN-mediated plant resistance. At least nine types of hormones have been identified in plants, including auxins, cytokinins (CK), gibberellins, abscisic acid, ET, brassinosteroids, SA, JA, and strigolactones (Su *et al.*, 2017). Among these, SA, JA, and ET are well known for their regulatory roles in plant defense against pathogens and thus are usually described as immunity-related hormones (Ma and Ma, 2016; Akhtar *et al.*, 2020). The other hormones, which are traditionally considered as growth hormones (e.g. auxins, CK, gibberellins, and brassinosteroids), have also been shown to be involved in plant–pathogen interactions (Ma and Ma, 2016; Chanclud and Morel, 2016). Interestingly, contrasting roles (positive or negative) in plant defense have been reported for CK, in part depending on the dose involved (Albrecht and Argueso, 2017; Spallek *et al.*, 2018; Akhtar *et al.*, 2020). CK signaling for leaf growth has been shown to be dependent on CWIN activity (Lara *et al.*, 2004). However, the reverse is not necessarily the case. For instance, an elevation of CWIN activity in tomato delayed leaf senescence but with no impact on the CK level in leaves (Jin *et al.*, 2009). It remains unknown whether and how CWIN-mediated plant–pathogen interaction involves CK signaling. Similarly, while the application of ET could affect CWIN gene expression or activity (Linden *et al.*, 1996; Sun *et al.*, 2021), it is unknown whether changes in CWIN activity or expression may affect the ET level and signaling during defense against pathogens.

**Fig. 1.**
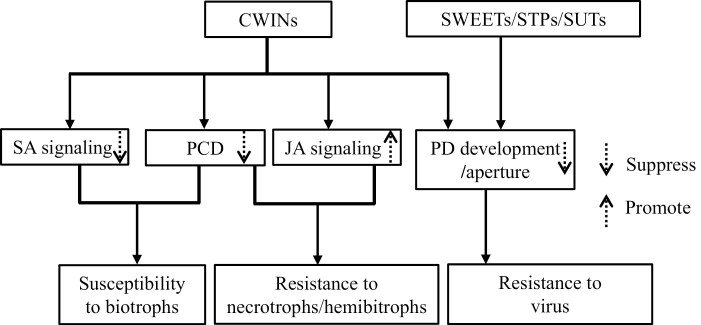
A hypothetical model illustrating how cell wall invertase (CWIN) and sugar transport protein (STP) differentially regulate plant resistance to different pathogens. Plant CWINs inhibit programmed cell death (PCD) and salicylic acid (SA) signaling, and simultaneously promote jasmonic acid (JA) signaling, which collectively contributes to susceptibility to biotrophic pathogens but increases plant resistance to necrotrophic/hemibiotrophic pathogens. Similar to biotrophic fungi and bacteria, viruses also require living tissues. However, in response to viral infection, CWINs and sugar transporters (SWEETS, STPs, and SUTs) appear to enhance resistance to viruses, possibly through inhibiting the formation or opening of plasmodesmata (PD) via modulating callose deposition around PD.

Generally, the SA-mediated signaling pathway promotes plant resistance to biotrophic pathogens, whereas the JA/ET pathway enhances resistance to necrotrophic/hemibiotrophic pathogens (Thaler *et al.*, 2004). There is an antagonistic relationship between the SA- and JA-mediated defense signaling pathways, since SA and JA have opposing influences on the expression of many defense genes (Glazebrook, 2005). Here, CWIN appears to interact negatively with SA but positively with JA signaling. For instance, in the maize mutant *miniature*, which shows the loss of CWIN activity in grain, the SA level was increased 10-fold compared with the wild-type maize (LeClere *et al.*, 2008). Similarly, the inhibition of CWIN activity by the application of acarbose, a chemical INV inhibitor, led to increased SA levels in Arabidopsis (Bonfig *et al.*, 2010). On the other hand, silencing of the CWIN gene *LIN5* resulted in a reduction in JA levels in tomato (Zanor *et al.*, 2009). The different responses of SA and JA to CWIN may explain why CWIN has contrasting effects on plant resistance to pathogens with different lifestyles. CWIN may reduce plant resistance to biotrophic pathogens possibly through inhibiting the SA signaling pathway but increase the resistance to necrotrophic/hemibiotrophic pathogens by activating the JA signaling pathway (Fig. 1). To date, however, studies on the roles of CWIN in plant–pathogen interactions have not reported potential changes in SA and JA levels (Essmann *et al.*, 2008; Kocal *et al.*, 2008; Siemens *et al.*, 2011; Sun *et al.*, 2014), whereas those investigating the impact of CWIN on SA and JA levels were conducted outside the context of plant–pathogen interaction (LeClere *et al.*, 2008; Zanor *et al.*, 2009). Clearly, future efforts are needed to experimentally test the CWIN–SA/JA hypothesis during plant–pathogen interactions.

Second, CWIN may indirectly affect plant–pathogen interactions through impacting on the incidence of programmed cell death (PCD). PCD is often induced during plant–pathogen interactions as part of the defense response (Gilchrist, 1998). There is compelling evidence that CWIN-mediated Suc degradation has important roles in the regulation of PCD. For example, the loss of function of CWIN in the maize mutant *mn1* led to PCD in the placento-chalazal region of the kernel (Kladnik *et al.*, 2004). Consistently, an elevation of CWIN activity achieved through silencing its inhibitor blocked heat-stress-induced PCD, thereby alleviating tomato fruit abortion (Liu *et al.*, 2016), while increased CWIN activity achieved through the injection of Suc into stems sustained grain set in maize under drought conditions, again through, in part, reducing the incidence of PCD (Boyer and McLaughlin, 2007). It is well known that PCD impairs the development of biotrophic pathogens by blocking their colonization of living cells, but benefits that of necrotrophic/hemibiotrophic pathogens (Greenberg, 1997). Thus, it is plausible to hypothesize that the inhibition of PCD by CWIN could favor or block biotrophic and necrotrophic/hemibiotrophic pathogen infections, respectively (Fig. 1).

## SWEETs: susceptibility or resistance factors in plant–pathogen interactions?

CWIN-mediated Suc metabolism and signaling are tightly coupled with sugar transport across cell membranes for plant development and defense (Ruan, 2014; Gebauer *et al.*, 2017; Liao *et al.*, 2020). Thus, we next explored how sugar transport may modulate plant defense response by focusing on three main sugar carriers (Doidy *et al.*, 2012; Pommerrenig *et al.*, 2020): SWEET, STP/MST (Sugar Transport Protein/MonoSaccharide Transporter), and SUT/SUC (SUcrose Transporter/SUcrose Carrier).

SWEETs passively transport Suc and/or hexose along a sugar concentration gradient, and thus function as bidirectional transporters, to exert effects on multiple physiological functions such as seed filling, phloem loading and unloading, nectar secretion, and plant–pathogen interaction (Chen *et al.*, 2012; Eom *et al.*, 2015; Jeena *et al.*, 2019). SWEETs are phylogenetically classified into four clades (Chen *et al.*, 2010; Chandran, 2015). Among them, Clades I and II exhibit a preference for Glc over Fru, while Clades III and IV predominantly transport Suc and Fru, respectively (Zhao *et al.*, 2018). Apart from Clade IV SWEETs operating on tonoplasts, the other three classes of SWEETs mainly function on PMs (Breia *et al.*, 2021). During infection, SWEETs generally facilitate the export of Suc and/or hexose out of host cells, which increases sugar availability to pathogens in the apoplasm (L.Q. Chen *et al.*, 2015; Pommerrenig *et al.*, 2020; Breia *et al.*, 2021). SWEETs thus act as susceptibility factors in this context.

Early evidence on the roles of SWEETs in plant–pathogen interactions mainly came from studies on Clade III members in rice (Li *et al.*, 2013; Streubel *et al.*, 2013). Through direct binding of secreted effectors to the promoters of SWEET genes, the hemibiotrophic bacterial pathogen Xoo PXO99^A^ hijacked and induced the expression of *OsSWEET11* and *OsSWEET14* in rice, both of which belong to the Clade III PM-located SWEETs; this promoted the virulence of Xoo by facilitating Suc efflux into the apoplasm for uptake by the pathogen. Accordingly, RNA interference (RNAi) repression of *OsSWEET11* increased the resistance of rice to Xoo (Chen *et al.*, 2010). As well as being associated with susceptibility to bacterial pathogens, *OsSWEET11* also acts as a susceptibility gene in rice infected by the necrotrophic fungal pathogen *Rhizoctonia solani* (Gao *et al.*, 2018). Similarly, in cotton, silencing of *GhSWEET10*, an ortholog of *OsSWEET11*, enhanced resistance to the hemibiotrophic bacterial blight pathogen *Xanthomonas citri* subsp. *malvacearum* (Xcm), most likely owing to reduced apoplasmic supply of Suc to Xcm, since the overexpression of *GhSWEET10* in *Nicotiana benthamiana* increased the concentration of Suc in the apoplasmic fluid of the transgenic leaves (Cox *et al.*, 2017). In addition, the phloem-localized Clade III transporters AtSWEET11 and AtSWEET12 act as susceptibility factors in Arabidopsis during infection by the fungal hemibiotroph *Colletotrichum higginsianum*, since *sweet11/sweet12* double mutants showed increased resistance toward this pathogen (Gebauer *et al.*, 2017). Apart from Clade III SWEETs, other clades of SWEETs also play negative roles in plant–pathogen interactions. For example, knockout of *AtSWEET4*, which encodes a Clade II Glc transporter, increased the resistance of Arabidopsis to necrotrophic *B. cinerea*, implying that AtSWEET4 may benefit fungal growth through facilitating the acquisition of hexose from the host cells by the pathogen (Chong *et al.*, 2014).

It is worth noting that some *SWEET*s could also function as resistance genes. In Arabidopsis, mutation of *AtSWEET2*, encoding a Clade I SWEET, resulted in increased susceptibility to the root necrotrophic pathogen *Pythium irregulare* (H.Y. Chen *et al.*, 2015). This is not surprising, since AtSWEET2 is responsible for the sequestration of Glc into vacuoles in Arabidopsis roots, as shown by the finding that the *Atsweet2* mutant displayed increased Glc efflux from the vacuole into the cytoplasm and subsequent increased exudation of Glc into the rhizosphere for uptake by *P. irregulare* (H.Y. Chen *et al.*, 2015). Intriguingly, silencing of PM-localized *IbSWEET10*, a Clade III SWEET, resulted in susceptibility to the hemibiotroph *Fusarium oxysporum* f. sp. *batatas* in sweet potato, while its overexpression increased resistance (Li *et al.*, 2017). These findings are clearly in contrast to those in rice and cotton (Chen *et al.*, 2010; Cox *et al.*, 2017), in which Clade III SWEETs contributed to susceptibility.

In Arabidopsis, the Clade III SWEETs AtSWEET11 and AtSWEET12 are specifically expressed in phloem parenchyma cells and xylem vessel-associated cells of floral stems (Chen *et al.*, 2012; Le Hir *et al.*, 2015). The double mutant of AtSWEET11 and AtSWEET12 showed not only a reduced area of both phloem and xylem poles in the floral stem, but also changes in the chemical composition of cell walls in vascular tissue, including reduced pectin and cellulose content, due to impaired sugar delivery from phloem to the adjacent vascular tissues (Le Hir *et al.*, 2015). Considering that plants usually strengthen the cell wall as a defense strategy upon pathogen infection through the deposition of wall chemicals including callose, pectin, and lignin (Rodriguez-Galvez and Mendgen, 1995), this finding from Arabidopsis (Le Hir *et al.*, 2015) may help us to understand why IbSWEET10, an ortholog of AtSWEET11 or AtSWEET12, could act as a resistance factor in sweet potato roots against the vascular pathogen *F. oxysporum*, which usually penetrates the root vasculature and then spreads upward (Keane, 2012). It is possible that the down-regulation of IbSWEET10 could compromise cell wall integrity in the vascular tissues by reducing the deposition of pectin and cellulose, leading to reduced resistance to *F. oxysporum*. Analyses of stem cross sections showed that *IbSWEET10*-RNAi lines exhibited a destroyed pith structure after infection, which was not observed in the wild-type plants (Li *et al.*, 2017). Studies on the role of SWEETs in plant–pathogen interaction were largely carried out with hemibiotrophs/necrotrophs, and little is known about the potential roles of SWEETs in response to infection with biotrophs (Table 2). Furthermore, the reported studies were mostly conducted on Clade III SWEETs, and there is much less information on the involvement of SWEETs from the other clades in pathogenicity. Thus, there is huge potential to explore along these lines.

**Table 2.** Roles of SWEETs in defense are coupled with the ways in which pathogenic microbes absorb nutrients

Pathosystem	Sugar-absorbing site	Sugar transporter	Role in defense	Function of SWEETs	Reference
**Necrotrophic/hemibiotrophic fungi**					
*Botrytis cinerea/*Arabidopsis leaf	Apoplasm	AtSWEET4 (Clade II, PM)	Negative	Glc exporter	Chong *et al.* (2014)
*Rhizoctonia solani/*rice sheath	Apoplasm	OsSWEET11 (Clade III, PM)	Negative	Suc exporter	Gao *et al.* (2018)
*Colletotrichum higginsianum /*Arabidopsis leaf	Apoplasm	AtSWEET11/12 (Clade III, PM)	Negative	Suc exporter	Gebauer *et al.* (2017)
*Fusarium oxysporum/*sweet potato root	Apoplasm (vascular vessel)	IbSWEET10 (Clade III, PM)	Positive	Suc exporter	Li *et al.* (2017)
*Pythium irregulare/*Arabidopsis root	Apoplasm	AtSWEET2 (Clade I, tonoplast)	Positive	Glc importer	H.Y. Chen *et al.* (2015)
**Hemibiotrophic bacteria**					
*Xanthomonas oryzae* pv. *oryzae* (Xoo)/rice leaf	Apoplasm	OsSWEET11/13/14 (Clade III, PM)	Negative	Suc exporter	Chen *et al.* (2010); Zhou *et al.* (2015); Blanvillain-Baufumé *et al.* (2017)
*Xanthomonas citri* subsp. *malvacearum* (Xcm)/cotton leaf	Apoplasm	GhSWEET10 (Clade III, PM)	Negative	Suc exporter	Cox *et al.* (2017)

PM, Plasma membrane.

## Elusive roles of STPs in plant–pathogen interactions

In most cases, the infected regions of plant tissues accumulate soluble sugars (Kocal *et al.*, 2008). However, pathogen-induced expression of *CWIN*s and *SWEET*s is generally not accompanied by an increase in apoplasmic sugar content (Yamada *et al.*, 2016). One possible explanation is that the *STP*s are up-regulated in parallel to retrieve apoplasmic sugars back into the host cells (Fotopoulos *et al.*, 2003; White and Frommer, 2015; Ding and Jones, 2017).

The STPs characterized thus far are all PM-localized H^+^/hexose symporters that facilitate energy-dependent hexose import into plant cells (Doidy *et al.*, 2012; Pommerrenig *et al.*, 2020). When the apoplasmic concentration of Glc elicited by the flg22 peptide of bacterial flagellin was compared in the leaves of wild-type Arabidopsis plants and the *stp1stp13* double mutant, a higher concentration of Glc was observed in the double mutant, which also exhibited an aggravated susceptibility to the bacterium *Pst* DC3000 (Yamada *et al.*, 2016), demonstrating a role of STP in reducing the apoplasmic sugar level and in conferring pathogen resistance. Further, the overexpression of *AtSTP13* enhanced Arabidopsis resistance to the necrotrophic fungus *B. cinerea*, whereas the mutation of *AtSTP13* resulted in the opposite effect, implying that STP13 may improve resistance by fueling the plant defense response and depriving the fungus of sugar nutrients (Lemonnier *et al.*, 2014).

**Fig. 2.**
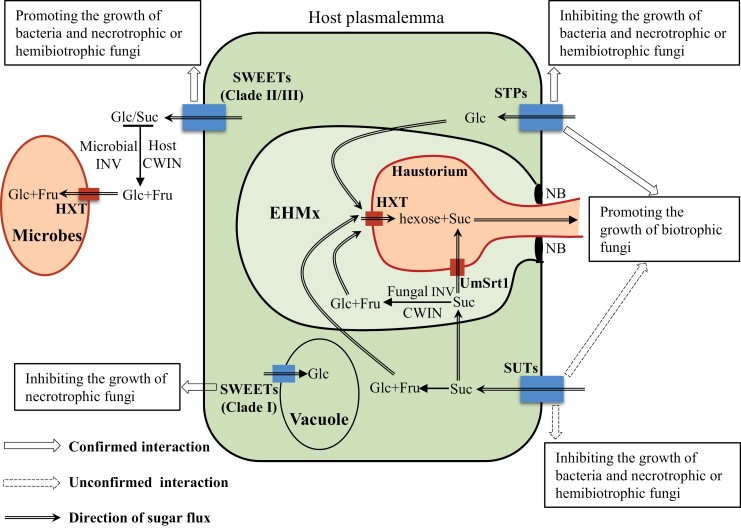
A schematic model of the different roles of plant sugar transporters in response to pathogen infection and the involvement of microbial INV and sugar transporters in plant–pathogen interactions. Clade II and III SWEETs export Glc and Suc into the plant apoplasm, respectively. Glc is then directly taken up into bacteria and necrotrophic/hemibiotrophic fungi during the initial infection phase via their own plasmalemma-localized hexose transporters (HXT), whereas Suc is first hydrolyzed into Glc and Fru by plant CWIN or pathogen-secreted INV before being imported into the pathogen cells. Consequently, these SWEETs promote bacterial and necrotrophic/hemibiotrophic fungal growth in the apoplasm. By contrast, Clade I SWEETs can sequester cytosolic Glc into plant cell vacuoles, thereby reducing the availability of Glc in the apoplasm, which starves necrotrophic fungi. The plant STPs facilitate hexose uptake into plant cells, which is subsequently released into the extrahaustorial matrix (EHMx) for uptake by biotrophic fungi via fungal HXT, thus promoting fungal infection. On the other hand, STPs reduce the concentration of Glc at the apoplasm, which inhibits the development of bacteria and necrotrophic/hemibiotrophic fungi. Plant SUTs take up apoplasmic Suc into plant cells and are commonly induced by pathogen infection. Studies in mycorrhizal fungi indicate that SUT (i) may promote the development of biotrophic fungi through increasing the intracellular sugar pool for subsequent import to the EHMx and uptake by Suc transporters such as UmStrt1, and (ii) could inhibit the development of bacteria and necrotrophic/hemibiotrophic fungi by limiting Suc availability in the apoplasm of the plant cell. NB, neckband.

However, STPs could also play a negative role in defense against biotrophic fungi. For example, Lr67res, a protein derived from the mutation of its wild-type version Lr67sus (an STP13 homolog from wheat) in two amino acids (Arg144Gly and Leu387Val), showed loss of Glc uptake activity in yeast cells (Moore *et al.*, 2015). Wheat lines expressing Lr67res showed a broad-spectrum resistance to biotrophic fungi including rust pathogens (i.e. leaf rust *Puccinia triticina*, stripe rust *Puccinia striiformis*, and stem rust *Puccinia graminis*) and the powdery mildew pathogen *B. graminis*. This broad resistance potentially resulted from the triggering of a plant defense response via increased sugar signaling due to Glc accumulation in the leaf apoplasm (Moore *et al.*, 2015). A recent study from the same team further showed that ectopic overexpression of wheat *Lr67res* in barley increased resistance to leaf rust (*Puccinia hordei*) and powdery mildew (*B. graminis*) due to higher expression of PR genes (Milne *et al.*, 2019). Consistently, knockdown of *TaSTP6*, another STP member in wheat, increased resistance to the rust pathogen *P. striiformis*, whereas the ectopic expression of *TaSTP6* in Arabidopsis increased plant susceptibility to powdery mildew (Huai *et al.*, 2019). Overall, these findings indicate that plant STPs generally promote the proliferation of biotrophic fungi during infection (Fig. 2; Table 3).

**Table 3.** Roles of hexose importer STPs in defense are dependent on the ways in which pathogenic microbes acquire nutrients

Pathosystem	Sugar-absorbing site	Sugar transporter	Role in defense	Reference
**Necrotrophic/hemibiotrophic fungi**				
*Botrytis cinerea*/Arabidopsis leaf	Apoplasm	AtSTP13	Positive	Lemonnier *et al.* (2014)
**Bacteria**				
*Pst* DC3000/Arabidopsis leaf	Apoplasm	AtSTP1/13	Positive	Yamada *et al.* (2016)
**Biotrophic fungi**				
Rust (*Puccinia triticina*, *Puccinia striiformis*, *Puccinia graminis*) and powdery mildew (*Blumeria graminis*)*/w*heat leaf	EHMx	TaSTP13	Negative	Moore *et al.* (2015)
Rust (*P. hordei*) and powdery mildew (*B. graminis*)*/*barley leaf	EHMx	TaSTP13	Negative	Milne *et al.* (2019)
Stripe rust (*P. striiformis*)*/*wheat leaf	EHMx	TaSTP6	Negative	Huai *et al.* (2019)
**Virus**				
Tomato yellow leaf curl virus/tomato leaf	N/A	LeHT1	Positive	Eybishtz *et al.* (2010)

EHMx, Extrahaustorial matrix; N/A, not applicable; PM, plasma membrane.

## Possible involvement of SUTs in plant–pathogen interactions

Most SUTs function as H^+^-coupled symporters to move Suc across the PM into the cytosol (Doidy *et al.*, 2012). Similar to STPs, the expression of SUTs is also generally up-regulated by pathogen infection, for example, in maize infected by *Colletotrichum graminicola* (Vargas *et al.*, 2012) and melon infected with cucumber mosaic virus (Gil *et al.*, 2011). However, the exact roles of SUTs in plant–pathogen interactions remain unclear. It was proposed that SUTs are unlikely to be the main transporters responsible for the retrieval of apoplasmic Suc due to (i) the energy-expensive but seemingly futile cycle of Suc uptake and release brought about by SUTs and SWEETs, and (ii) their low affinity for Suc and acidic optimum pH (pH 5–6), considering the alkalized apoplasmic environment (pH >6) during infection (see Pommerrenig *et al.*, 2020). However, a study on arbuscular mycorrhizal fungi indicated that transgenic tomato plants with reduced expression of *SlSUT2* exhibited increased mycorrhization owing to the weakened retrieval of Suc from the apoplasm back to the host cells (Bitterlich *et al.*, 2014); this implies that SUTs may act like STPs to regulate plant–pathogen interactions by modulating the availability of Suc in the host apoplasm.

## The roles of sugar transporters in defense are dependent on the ways pathogens obtain sugars

It is clear that sugar transporters influence the outcome of plant–pathogen interactions primarily through mediating the allocation of sugars between host plants and pathogens (Huai *et al.*, 2019). Delivery of sugars to the absorbing site of pathogens would inevitably benefit their proliferation. Bacteria (regardless of their lifestyle) and necrotrophic/hemibiotrophic fungi mainly take up sugar from the host apoplasmic space (Lemoine *et al.*, 2013; Xin *et al.*, 2016). Thus, it is not surprising that plant sugar importers (STPs and Clade I SWEETs) and exporters (Clade II and III SWEETs) act as resistance and susceptibility factors, respectively, during infection by bacteria and necrotrophic/hemibiotrophic fungi through reducing or increasing the availability of sugars in the apoplasm accordingly (Tables 2, 3, Fig. 2).

Unlike pathogenic bacteria and necrotrophic/hemibiotrophic fungi, biotrophic fungi such as powdery mildew, rust fungi, and the corn smut fungus *Ustilago maydis* acquire sugar not from the host cell apoplasm but from a specialized apoplasm named the extrahaustorial matrix (EHMx) via haustoria (Roberts *et al.*, 1993; Wahl *et al.*, 2010; Chang *et al.*, 2017). The EHMx is bordered by the haustorial membrane and host PM and is separated from the bulk apoplasm by the physical fusion of both membranes at the ‘neckband’ (Chang *et al.*, 2017; Fig. 2). Sugar must be first transported into host cells and then released into the EHMx for uptake by the pathogen (Bezrutczyk *et al.*, 2018). In this scenario, plant PM-localized sugar importers (STPs) may act as susceptibility factors to biotrophic fungi through facilitating the accumulation of sugars in the intracellular space of infected host cells, from which sugars are subsequently exported into the EHMx for uptake by the pathogen (Table 3, Fig. 2). This model explains why sugar-importer STPs act as resistance factors to bacteria and necrotrophic/hemibiotrophic fungi, but as susceptibility factors to infection by biotrophic fungi.

Overall, the available evidence shows that the specific roles of sugar transporters in plant–pathogen interactions, and particularly in sugar partitioning between pathogens and host cells, are dependent on their cellular locations. If they help pathogens gain sugar nutrients, these sugar transporters act as susceptibility factors. Otherwise, they contribute to resistance, as indicated in the model depicted in Fig. 2. This model accommodates almost all available published studies, with one exception, in which the silencing by RNAi of IbSWEET10, a putative PM-localized Clade III Suc exporter, reduced, rather than increased, resistance of sweet potato to the hemibiotrophic fungus *F. oxysporum* (Li *et al.*, 2017; Table 2), which resides in vascular tissues (Yadeta and Thomma, 2013). The decreased resistance could be explained by the blocked development of vasculature in the RNAi plants, as revealed by examination of stem cross sections (Li *et al.*, 2017), possibly due to reduced sugar delivery from the phloem to the surrounding vascular tissue as a result of decreased SWEET expression (Le Hir *et al.*, 2015). Although one cannot exclude the possibility that the RNAi-mediated silencing may also inhibit the growth of *F. oxysporum*, the available evidence suggests that the decreased resistance in the RNAi plants more likely results from compromised vascular development in the host than an effect on the fungal growth. It remains to be verified whether this is the case and whether it is a general phenomenon for SWEETs to act as resistance factors in plant defense against vascular pathogens.

## Sugar-mediated plant responses to viral infection

The plant response to viral infection exhibits some similarities to plant–bacterial and plant–fungal interactions, as well as certain differences. For instance, apoplasmic expression of yeast-derived INV increased tobacco resistance to potato virus Y (Herbers *et al.*, 1996). Pertinently, silencing of the gene encoding the hexose/H^+^ symporter LeHT1, a PM-located STP in tomato (McCurdy *et al.*, 2010), increased susceptibility to tomato yellow leaf curl virus (Eybishtz *et al.*, 2010; Sade *et al.*, 2013). These cases suggest positive roles of CWINs and STPs in resistance to virus infection (Tables 1, 3). Like biotrophic fungi, viruses also require living tissue for spread and replication and can be considered as obligate biotrophs (Shapiro *et al.*, 2013; Gullner *et al.*, 2017). However, these findings do not appear to fit the hypothesis discussed previously that CWINs and STPs promote the growth of biotrophic fungi and bacteria (Tables 1, 3). In contrast to pathogenic bacteria, fungi, and oomycetes, which mainly reside in the intercellular space, viruses replicate within the cytoplasm of the host cells and spread from cell to cell via plasmodesmata (PD)—intercellular and membrane-lined cytoplasmic channels (Eybishtz *et al.*, 2010; Nassem *et al*., 2017). In this scenario, it is unlikely that STPs would block virus infection by sequestering sugars to ‘starve’ the virus, as they do when dealing with fungal and bacterial infection. Thus, different mechanisms must be employed by CWINs and STPs to mediate resistance to viruses.

Several studies indicate an inverse relationship between PD opening (the symplasmic pathway) and the expression/activity of sugar transporters. For example, in single-celled cotton fibers, there was little or no expression of GhSUTs and Clade III GhSWEETs when PD were open early in elongation, whereas increased expression of these transporters was observed when PD were closed during the late stage of fiber elongation (Ruan *et al.*, 2001; Zhang *et al.*, 2017). Furthermore, callose-induced closure of PD led to an increased and prolonged expression of GhSUTs and Clade III GhSWEETs (Zhang *et al.*, 2017). An inverse correlation has also been observed between CWIN activity and PD gating. During fruit development of Chinese jujube, for instance, phloem unloading occurs apoplasmically in both the early and the late stage, but symplasmically in the middle phase, which correlates with high, low, and high CWIN activity in the respective stages (Nie *et al.*, 2010). Evidence from transgenic studies also supports this inverse relationship in other systems. The ectopic expression of yeast INV in the apoplasm of tobacco led to the arrest of PD development in mature leaves (Ding *et al.*, 1993). Thus, it appears that higher expression of CWINs and STPs may block virus infection by disrupting PD development or function, which could explain why CWIN and STP play positive, rather than negative, roles in plant resistance to viruses (Fig. 1). The molecular basis underlying CWIN- or STP-mediated regulation of PD in response to viral infection remains to be determined. One possibility is that enhancing CWIN and STP activity could generate more Glc for callose deposition to block the PD aperture and hence the cell-to-cell spread of virus.

## The forgotten side: roles of microbial INV and sugar transporters

The final outcome of plant–pathogen interaction is determined by factors from both sides. However, studies on the roles of sugars in the response to pathogen attack have mostly focused on the host, with little attention being paid to the pathogen in terms of how the latter may manipulate its own sugar uptake and metabolic systems to win the ‘tug of war’ with the host for sugar resources.

Most pathogens absorb sugars from the host apoplasm in the form of hexose (mainly Glc) rather than Suc (Tetlow and Farrar, 1992; Bisson *et al.*, 1993; Sutton *et al.*, 1999; Talbot, 2010; Veillet *et al.*, 2016; Julius *et al.*, 2017). To facilitate the utilization of SWEET-exported Suc, many pathogenic microbes secrete INV into the host cell wall to hydrolyze Suc into hexose, which is then taken up by the pathogen through its own hexose transporters (HXTs) (Parrent *et al.*, 2009). The INV-encoding gene in biotrophic pathogens was first identified in the rust fungus *Uromyces fabae* (Voegele *et al.*, 2006), in which Suc derived from the host cells is hydrolyzed by the rust INV, INV1p, in the EHMx, followed by the uptake of the resultant Glc and Fru by fungal HXT localized in the haustoria (Voegele *et al.*, 2001; Voegele and Mendgen, 2011). The fungal biotroph *P. striiformis*, the causal agent of wheat stripe rust, secretes abundant INV (PsINV) into the host apoplasm during its invasion; silencing of the *PsINV* gene inhibited the growth of the fungus, hence reducing its virulence (Chang *et al.*, 2017). Given that *P. striiformis* expresses only HXT and not a Suc transporter (Cantu *et al.*, 2011; Zheng *et al.*, 2013), it can be inferred that PsINV plays a major role in the pathogenicity of the fungus by hydrolyzing Suc into hexose for uptake by the fungal HXT (Chang *et al.*, 2017).

Genes encoding INV and HXT are also expressed in necrotrophic/hemibiotrophic fungi. For example, the necrotroph *B. cinerea* has one extracellular INV gene and three HXT genes, which are induced during infection of Arabidopsis (Veillet *et al.*, 2016). Five HXTs (CgHXT1–5) have also been identified in the hemibiotrophic fungus *C. graminicola*, which causes stem rot and leaf anthracnose in maize. Among them, the expression of CgHXT1 and CgHXT3 was induced at the biotrophic stage of *C. graminicola* infection, whereas CgHXT2 and CgHXT5 were up-regulated at the necrotrophic stage (Lingner *et al.*, 2011). In some cases, induced extracellular INV activity in the infected plant tissue is entirely attributable to the necrotrophic fungus rather than the host plant (Jobic *et al.*, 2007; Box 1).

Similar to fungi, many pathogenic bacteria also express extracellular INV and sugar transporters (Ziegler and Albersheim, 1977; Vásquez-Bahena *et al.*, 2006). However, bacteria appear very different from their fungal counterparts in their mode of obtaining sugars from the host. Some transcriptome analyses indicate no induction of bacterial INVs and sugar transporters during invasion of host plants, for example, in the interactions of *Dickeya dadantii* and Arabidopsis, and *Xanthomonas axonopodis* and soybean (Chapelle *et al.*, 2015; Chatnaparat *et al.*, 2016). These observations suggest that pathogenic bacteria may obtain sugars for their growth not through changing their own Suc degradation and sugar transport system, but possibly through modifying or hijacking the counterparts of the host cells. For instance, the pathogenic bacterium Xcv secretes T3SS-dependent effector (XopB) to block the induction of host CWIN, enhancing its virulence (Sonnewald *et al.*, 2012). In rice, as discussed earlier, Xoo secretes T3SS-dependent effector (PthXo1) to bind the promoters of the Clade III SWEET genes *OsSWEET11* and *OsSWEET14* to activate their transcription, resulting in the provision of Suc to fuel the infection (Chen *et al.*, 2010). Indeed, the type III secretion mutant (ΔhrcU) of *Pst* DC3000 failed to induce the expression of three *AtSWEET*s in Arabidopsis, resulting in reduced pathogenicity (Chen *et al.*, 2010).

There are few pathogens that can directly take up and utilize Suc as their major C source without the need for degradation of Suc into hexose by plant and/or microbial INV in the apoplasm. The biotrophic fungus *U. maydis*, which causes corn smut disease in maize, expresses a Suc-specific sugar transporter, UmSrt1, which is PM localized and expressed only after successful invasion of the maize tissues (Wittek *et al.*, 2017) (Fig. 2). UmSrt1 shows a higher affinity for Suc than the maize Suc transporter ZmSUT1 and thus possibly outcompetes ZmSUT1 during the battle for limited intercellular Suc (Wahl *et al.*, 2010; Wittek *et al.*, 2017). Deletion of *UmSrt1* using a PCR-based gene replacement system strongly reduced the virulence of *U. maydis*, indicating a central role of the fungal protein in the maize–*U. maydis* interaction (Wahl *et al.*, 2010). For Suc-favoring pathogens, it might be advantageous for them to take up Suc as a C source, as this step bypasses Suc hydrolysis, thus potentially blocking the activation of plant defense responses, since it is generally the hexose, and not Suc, that triggers the plant defense response (Wahl *et al.*, 2010). In this situation, manipulation of the host’s CWIN activities may differentially affect the proliferation of Suc-favoring or hexose-favoring pathogens through modulating the availability of apoplasmic Suc and hexose. It is clear that more investigations into the mode of action of microbial CWINs and transporters are necessary for better understanding of how sugar metabolism and transport on each side determine the final outcome of plant–pathogen interactions.

## Conclusions and future directions

Our analyses showed that the specific regulation of plant–microbe interactions by CWIN, SWEET, and STP are conditioned by the given pathosystem involved. Here, the roles of CWIN in the host response to pathogen attack are largely dependent on the lifestyle of pathogenic fungi and bacteria. Similarly, the nature of SWEET/STP-mediated plant–pathogen interactions varies, depending on the cellular sites from which pathogens acquire sugar nutrients. Furthermore, CWIN and SWEET/STP modulate plant defense against viral spread through, at least in part, impacting on PD formation or aperture. Finally, we draw attention to the great need for research on the pathogen-originating CWINs and sugar transporters, as these microbial components could exert significant influence on the plant–pathogen interaction and thereby shape the final outcome of the interaction.

Looking ahead, although extensive studies have been performed to investigate how CWINs affect plant–pathogen interactions, little is known about effects of the other Suc-degrading enzymes (i.e. VIN, CIN, and Sus) on plant defense. It will also be of significance to determine whether it is a general phenomenon for SWEETs to act as resistance factors, instead of susceptibility factors, in defense against vascular pathogens (Li *et al.*, 2017) (Table 2). Equally, there is a lack of studies on the roles of SWEETs in plant–biotroph interactions, despite available information on their roles in responding to hemibiotrophs/necrotrophs (Table 2). Most notably, although sugar signaling has long been proposed to be involved in defense responses against pathogens (Rolland *et al.*, 2006; Wingler and Roitsch, 2008), the potential signaling roles of CWIN and sugar transporters in this complex interaction remain elusive. One challenge is to experimentally dissect their roles in signaling from that in the provision of C nutrients. Using reporter genes or sensors for sugar status coupled with tissue- or cell-specific expression analyses may be a promising approach to tackle this issue, as recently demonstrated in studies on the role of CWIN-mediated signaling in ovule initiation (Liao *et al.*, 2020) and on the control of cytosolic sugar homeostasis by tonoplast sugar transporters (Zhu *et al.*, 2021).

Plant defense against pathogen attack is further complicated by abiotic stresses such as drought, heat (Pandey and Senthil-Kumar, 2019), and increased CO_2_ concentration (Zhang *et al.*, 2015). The impact of these factors on defense depends on the pathogen involved and the frequency and the intensity of individual abiotic stresses that the host plants encounter. It is noteworthy that the effects of combined abiotic stresses on plant–pathogen interaction appear to be more dramatic than those of a single abiotic stress. For example, combined heat stress and drought made Arabidopsis plants more susceptible to infection by turnip mosaic virus compared with their susceptibility under either heat stress or drought alone (Prasch and Sonnewald, 2013). The elevation of CWIN activity by silencing its inhibitor gene (Jin *et al.*, 2009) activated the expression of PR genes in tomato ovaries (Ru *et al.*, 2017) and improved fruit set under heat stress (Liu *et al.*, 2016), indicating the potential for improving tolerance to both abiotic and biotic stresses by manipulating sugar metabolism and signaling. It remains to be elucidated how INVs and sugar transporters may regulate plant–pathogen interactions differently under abiotic stresses such as heat, cold, drought, or a combination of such stresses, compared with their regulatory roles under optimal conditions. With the increasing atmospheric CO_2_ concentration, global-warming-associated incidents of abiotic stresses are predicted to be more frequent and severe. It has thus become increasingly urgent to study plant–pathogen interactions in the context of abiotic stress, and such work will provide valuable insights for the improvement of crop performance in the face of climate change.

Finally, although beyond the scope of this review, it is recognized that sugar allocation is also central to plant defense against pests (Rehill and Schultz, 2003; Zinkgraf *et al.*, 2016). In this context, evidence from transgenic plants suggests that up-regulation of INV may enhance plant tolerance to insect herbivores. For instance, suppression of CWIN activity in tobacco compromised tolerance to simulated herbivory (Ferrieri *et al.*, 2015), with a similar phenotype observed in *VIN*- or *CIN*- knockout Arabidopsis mutants (Siddappaji *et al.*, 2015). More recently, *VST1*, encoding a phloem-expressed tonoplast transporter for the unloading of Suc and Glc in watermelon, was found to be induced by aphids, and loss of *VST1* via genome editing reduced aphid setting and honeydew production in young leaves of the mutant plants through blocking the supply of sugar in phloem sap to aphids (Li *et al.*, 2021, Preprint). Clearly, great potential exists to better understand sugar metabolism and transport for increasing resistance to not only pathogens but also pests.

Box 1. **Role of CWIN and sugar transporters in plant defense against pathogens**

- CWINs exert different roles in plant resistance to pathogens with different lifestyles

Overexpression of the CWIN gene *GIF1* in rice enhanced resistance to the hemibiotrophic bacterial pathogen *Xanthomonas oryzae* pv. *oryzae* (Xoo) and the hemibiotrophic fungal pathogen *Magnaporthe oryzae* through increases in cell wall thickness, reactive oxygen species accumulation, and the hypersensitive response, and activated the expression of PR genes including *PR1a*, *PR1b*, *PR3*, *PR10*, *WRKY45*, and *NPR1* (Sun *et al.*, 2014). However, silencing of the CWIN gene *LIN8* in tomato increased (rather than decreasing) leaf resistance to the biotrophic bacterial pathogen *Xanthomonas campestris* pv. *vesicatoria* (Xcv) (Kocal *et al.*, 2008). These findings indicate that CWINs may act as resistance and susceptibility factors in the response to hemibiotrophic and biotrophic pathogens, respectively (see Table 1 for more examples and Fig. 1 for possible mechanisms).

- Roles of STP in plant defense vary with the cellular sites from which pathogens acquire sugar nutrients

Mutation of *AtSTP13* resulted in reduced resistance of Arabidopsis to the necrotrophic fungus *Botrytis cinerea* (Lemonnier *et al.*, 2014). Similarly, double mutation of *AtSTP1/AtSTP13* in Arabidopsis increased susceptibility to the bacterial pathogen *Pst* DC3000 (Yamada *et al.*, 2016), implying that STP could enhance plant resistance by depriving bacteria and necrotrophic fungi of a supply of sugar in the apoplasm. In contrast, Lr67res, a mutated form of the wheat homolog (Lr67sus) of AtSTP13, was associated with a broad-spectrum resistance to biotrophic fungi including rust pathogens (*Puccinia triticina*, *Puccinia striiformis*, and *Puccinia graminis*) and the powdery mildew pathogen *Blumeria graminis* (Moore *et al.*, 2015). Furthermore, knockdown of *TaSTP6* increased wheat resistance to *P. striiformis*, whereas ectopic expression of *TaSTP6* in Arabidopsis reduced resistance to powdery mildew (Huai *et al.*, 2019). Unlike bacteria and necrotrophic/hemibiotrophic fungi, biotrophic fungi acquire sugars not from the cell wall apoplasm, but from specialized apoplasm named the extrahaustorial matrix (EHMx). Sugar must be first imported into host cells by STP and subsequently exported into the EHMx for uptake by biotrophic fungi (see Fig. 2 for details and Table 3 for more examples).

- CWIN and sugar transporters enhance resistance to viruses, possibly by inhibiting plasmodesmatal development or aperture

In contrast to bacteria and fungi, viruses spread from cell to cell within host plants through plasmodesmata (PD). In non-pathosystems, high expression of CWIN and sugar transporters commonly inhibits PD development or reduces PD aperture. For example, the ectopic expression of yeast INV in the apoplasm of tobacco leaves led to the arrest of PD development (Ding *et al.*, 1993) and reduced viral infection (Herbers *et al.*, 1996). Similarly, increased expression of GhSUTs and clade III GhSWEETs correlated with a reduction in PD aperture in cotton fiber (Zhang *et al.*, 2017). Thus, CWIN and sugar transporters may block virus spread in plants via the inhibition of PD development or opening.

- Microbial INVs and sugar transporters: the forgotten, yet important, players in shaping the outcome of plant–pathogen interactions

Similar to host plants, pathogenic microbes also possess their own sugar uptake and metabolic systems to facilitate their pathogenicity. For example, during the infection of sunflower by the necrotrophic fungus *Sclerotinia sclerotiorum*, the protein level of host CWIN decreased, whereas that of microbial acid INV increased, indicating the the rise of extracellular INV activity in infected plant tissue may mainly derive from the pathogen instead of the host plant (Jobic *et al.*, 2007). Furthermore, silencing of the *PsINV* gene of the biotrophic fungus *P. striiformis* inhibited fungal growth and reduced spore number and virulence (Chang *et al.*, 2017). For the biotrophic fungus *Ustilago maydis*, deletion of the Suc transporter UmSrt1, which is responsible for Suc uptake into the fungus, reduced the virulence of the pathogen (Wahl *et al.*, 2010; Wittek *et al.*, 2017). Thus, microbial CWINs and sugar transporters play major roles in pathogenicity and must be taken into account to achieve a holistic understanding of sugar-modulated plant–pathogen interactions.
